# Characterisation of the macular dystrophy in patients with the A3243G mitochondrial DNA point mutation with fundus autofluorescence

**DOI:** 10.1136/bjo.2007.131177

**Published:** 2008-01-22

**Authors:** P P Rath, S Jenkins, M Michaelides, A Smith, M G Sweeney, M B Davis, F W Fitzke, A C Bird

**Affiliations:** 1 Moorfields Eye Hospital, London, UK; 2 Retina Vitreous Consultants, Pittsburgh, PA, USA; 3 Institute of Ophthalmology, London, UK; 4 County Hospital, Hereford, UK; 5 Department of Molecular Neuroscience, Institute of Neurology, London, UK

## Abstract

**Introduction:**

The mitochondrial DNA A3243G point mutation is associated with a wide variety of systemic manifestations including a macular dystrophy. The characteristics of fundus autofluorescence (AF) in these patients are distinctive and have not been previously described.

**Methods:**

A complete history and ophthalmic examination, including fundus photography and autofluorescence imaging, was performed on twelve probands harbouring the A3243G point mutation.

**Results:**

Four patients had diabetes, 10/12 hearing loss, and 7/12 were visually symptomatic. A positive family history was present in 5/12. Fundus findings consisted of two primary phenotypes: discontinuous circumferentially oriented perifoveal atrophy (9/12) or an appearance consistent with pattern dystrophy (3/12). In both phenotypes pale deposits and pigment clumping were seen at the level of the retinal pigment epithelium, with occasional changes also noted outside the arcades and nasal to the optic nerve. Fundus AF imaging revealed decreased autofluorescence in areas of atrophy and increased AF of the pale subretinal deposits. In areas of the retina that appeared normal clinically, variable sized flecks of increased and decreased AF were present.

**Conclusions:**

The mitochondrial DNA A3243G point mutation can result in disease with a variable presentation. Fundus autofluorescence reveals a recognisable phenotype in most cases that is different from other macular dystrophies.

The single-point mutation of the mitochondrial DNA (mtDNA) at the 3243 position in the tRNA^Leu(UUR)^ gene leads to a wide variety of clinical manifestations. This mutation was originally described in association with Mitochondrial Encephalopathy, Lactic Acidosis and Stroke-like episodes (MELAS), and is now known to produce a variety of other clinical disorders, including maternally inherited diabetes and deafness (MIDD), cardiomyopathy, chronic progressive external ophthalmoplegia (CPEO), a pure myopathy, gastrointestinal dysmotility and renal failure.^1^^–^6 One explanation for this variability is thought to be the load of mutant mtDNA present in an individual, which varies both between individuals and from tissue to tissue within an individual.^7^ In general, a higher mutation load is associated with more severe disease. However, this generalisation does not always hold true, and it is possible that environmental factors and/or nuclear genetic influences may modulate disease manifestations.^6^ ^8^ ^9^

Ocular manifestations of mitochondrial diseases are well recognised, and in the original report by Reardon *et al* which revealed a new subtype of diabetes caused by a mtDNA mutation, three patients were found to have retinal pigmentary changes.^2^ ^10^ However, there were no fundus photographs of the changes described in this initial report.^2^ In 1995, Massin *et al* described the association of a bilateral macular pattern dystrophy with MIDD and further characterised this in a subsequent publication in 1999.^11^ ^12^ Since these reports, there have been several additional publications describing the macular dystrophy of MIDD and MELAS, both clinically and electrophysiologically.^13^^–^18

The use of the confocal scanning laser ophthalmoscope (cSLO) for imaging of macular diseases has been well described.^19^^–^25 In this study, we have characterised in detail the distinct fundus autofluorescence (AF) characteristics of the macular dystrophy associated with the A3243G mtDNA mutation.

## METHODS

All patients diagnosed as having a macular dystrophy due to the A3243G mtDNA mutation in the Medical Retina Clinic at Moorfields Eye Hospital between 1995 and 2001 were included in the study. Twelve patients in total were evaluated. Patient demographics, referring diagnosis, presence or absence of visual symptoms, duration of visual symptoms, general medical history including presence or absence of diabetes and deafness, maternal history of diabetes and deafness, and a detailed family history, were obtained for each patient. Best-corrected Snellen visual acuity and slit-lamp biomicroscopy findings were recorded. Colour fundus photographs and fundus autofluorescence imaging were performed. Patients were tested for the A3243G mutation based on the characteristics of their ophthalmic examination, regardless of whether or not there was a personal or family history of diabetes or deafness. The diagnosis of the A3243G mtDNA mutation was established with DNA testing of peripheral blood samples using previously described techniques.^26^

Fundus autofluorescence imaging was performed with a confocal scanning laser ophthalmoscope (cSLO, Zeiss, Jena, Germany or HRA, Heidelberg, Germany) using previously published techniques.^19^ ^20^ ^27^ Autofluorescence images were compared with colour photographs. The AF images of the patients with the A3243G mutation were also compared with images of patients with other maculopathies, including Stargardt macular dystrophy, R172W peripherin macular dystrophy, bull’s eye macular dystrophy, geographic atrophy from age-related macular degeneration and pattern dystrophy.

Patients who did not have a history of diabetes were tested with fasting blood glucose, where possible, according to the recommendations of the American Diabetic Association.^28^ Renal function tests were also performed when possible due to the association of renal dysfunction with the A3243G mutation.^29^

## RESULTS

Twelve patients were diagnosed as having the A3243G mutation between January 1995 and February 2001; in none had the diagnosis of a mitochondrial disorder been made prior to being seen by the authors. The characteristics of these patients at presentation are summarised in table 1. 

**Table 1:** Maternally inherited diabetes and deafness patient characteristics at presentation

Patient no.	Sex	Age	Initial diagnosis	Visual symptoms	Diabetes	Hearing loss	Maternal family history of diabetes	Maternal family history of hearing loss
1	F	48	Usher	No	Yes	Yes	No	No*
2	F	42	Central areolar choroidal sclerosis	Yes	No	No	No†	No
3	F	53	Non-specific maculopathy	Yes	No	Yes	No	Yes
4	F	47	Non-specific maculopathy	Yes	No	Yes	No	Yes
5	F	36	Non-specific maculopathy	Yes	No	Yes	Yes	No
6	M	65	Non-specific maculopathy	Yes	No	Yes	No	No*
7	F	43	Pattern dystrophy	No	No	Yes	?	?
8	M	43	Macular degeneration	Yes	Yes	Yes	No†	No
9	F	38	Non-specific maculopathy	Yes	No	Yes	No	No
10	F	55	Stargardt versus mitochondrial	No	No	Yes	No	Yes
11	F	48	Macular atrophy	No	Yes	No	Yes	Yes
12	M	46	Non-specific maculopathy	No	Yes	Yes	No	Yes
Total				7/12	4/12	10/12	2/12	5/12

*Hearing loss diagnosed after the age of 75; patients 3 and 4 are a mother and daughter, and ages are at presentation of disease.

†Adult onset DM diagnosed at age 60.

Nine of the patients were female. The average age at presentation was 47 years (range 36 to 65) with an average age at diagnosis of 51 (range 36 to 71). In six of 12 patients, the reason for referral to a specialist clinic was an unspecified maculopathy consisting of a combination of retinal pigment epithelial changes and atrophy. Other diagnoses at referral were Usher syndrome variant, central areolar choroidal sclerosis, pattern dystrophy, macular degeneration and macular atrophy. Five of the 12 patients did not have visual symptoms at presentation and were found to have fundus abnormalities on routine eye examination. Of the patients who were initially asymptomatic, two patients became symptomatic during review. Three patients are still asymptomatic after follow-up, ranging from 1 to 6 years. Asymptomatic patients tended to be younger than the patients with symptoms, with a mean age of 43 compared with 51 years of age, although the number of patients in each group is too small for statistical significance to be established. Of the seven patients with symptoms, three reported difficulties due to paracentral scotoma, and three reported a general decrease in vision in one eye.

A history of hearing loss was common, with 10 of 12 patients having some degree of hearing impairment. Patient 1 had a cochlear implant, and four other patients were using hearing aids. Diabetes was present in only four patients, all of whom also had hearing loss.

A maternal history of diabetes was present in two patients and a maternal history of deafness in five patients. A maternal history of both diabetes and deafness was elicited in only one subject. There were two additional patients with a maternal history of hearing loss diagnosed after the age of 75 years. This was considered to be age-related hearing loss and not included as a positive history in the context of a mitochondrial disease. Two further patients had a family history of maternal diabetes, which was not diagnosed until the age of 60, and was again not considered as a positive finding in the context of the A3243G mutation.

In all patients, presenting visual acuity was good, each having at least 6/9 or better vision, and seven patients achieving 6/6 or better vision, with each eye. At final follow-up, the vision was slightly worse, with all patients having 6/12 or better vision in at least one eye. Six out of 12 patients had 6/6 or better vision with at least one eye at the last documented examination. Of the four patients with 6/12 or worse vision, three had deterioration in vision due to paramacular atrophy encroaching upon fixation, and one had decreased vision bilaterally due to retinal pigment epitheliopathy.

**Figure 1:**
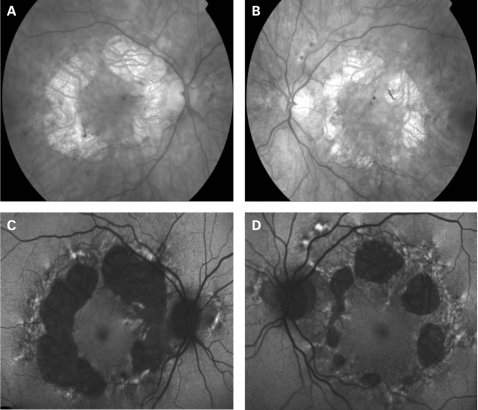
(A, B) Fundus photographs of patient 1 showing circumferential orientation and distribution of the discontinuous perifoveal atrophy. (C, D) Fundus AF images of patient 1 demonstrating decreased AF of the areas corresponding to chorioretinal atrophy and speckled AF surrounding the areas of atrophy. There is abnormal AF nasal to the optic nerve and just outside the superior temporal vascular arcade. Patient consent has been obtained for publication of this figure.

**Figure 2:**
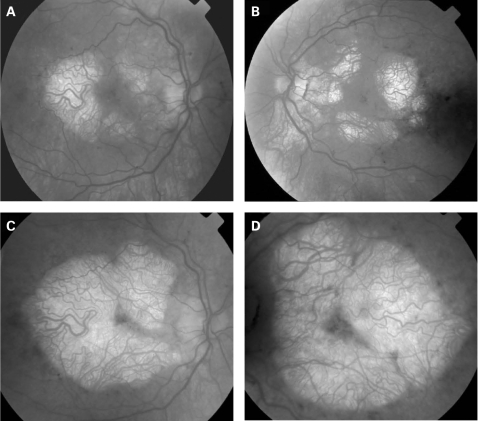
(A, B) Fundus photographs of patient 8 taken in 1994 showing circumferential distribution of the perifoveal atrophy. (C, D) Fundus photographs of patient 8 taken in 2001 showing that the atrophy has coalesced almost to a complete ring with central foveolar sparing. Patient consent has been obtained for publication of this figure.

**Figure 3:**
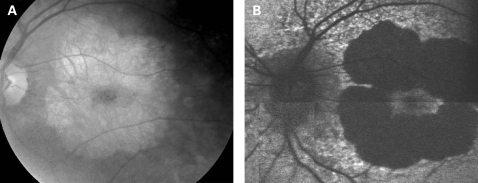
(A, B) Fundus photograph and AF image of patient 9 showing a large area of central atrophy corresponding to the decreased AF. There is extensive speckled AF surrounding the atrophy. This is the only patient who lost fixation due to progression of atrophic changes. Patient consent has been obtained for publication of this figure.

**Figure 4:**
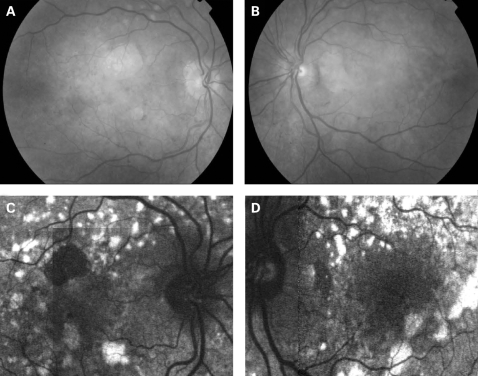
(A, B) Fundus photographs of patient 10 showing a granular appearance to the RPE. The yellow-white deposits at the level of the RPE are circumferentially oriented and extend just beyond the vascular arcades. The deposits are slightly more prominent in the left eye. In the right eye, there is a small circular patch of perifoveal atrophy present superiorly. (C, D) Fundus AF images of patient 10, 2 years after the fundus photographs. The pale yellow deposits are associated with increased AF oriented in a circular fashion surrounding the macula and optic nerve. There is decreased AF in areas of atrophy and a diffuse abnormality of AF within the macula and surrounding the optic nerve, which is greater than expected, based on the fundus photographs. Patient consent has been obtained for publication of this figure.

The fundus appearance was variable between patients, but two separate phenotypes were identified. The most common phenotype, occurring in nine of the 12 patients, was discontinuous perifoveal atrophy that was circumferentially distributed and oriented (fig 1). In patients who had follow-up over many years, the atrophy coalesced into a ring (fig 2). The central fovea was spared in all but one eye of a single patient (fig 3). Adjacent to the areas of atrophy were pale deposits at the level of the retinal pigment epithelium (RPE), granularity of the RPE and subretinal pigment clumping. The second phenotype, present in three patients, was an appearance consistent with a pattern dystrophy. In these three patients, no significant atrophy was present in the perifoveal area. There was granularity of the RPE and pale deposits and pigment clumping at the level of the RPE (fig 4). The majority of the fundus changes occurred within the temporal vascular arcades; however, RPE changes were also seen outside the arcades and nasal to the optic nerve.

**Figure 5:**
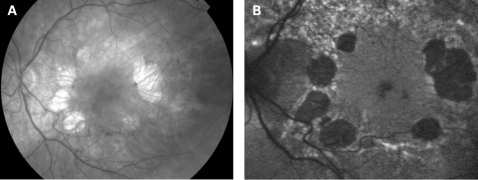
(A, B) Fundus photograph and AF image of patient 11. The areas of decreased AF correspond to the atrophy, which is circumferentially oriented and surrounded by irregular increased AF. Patient consent has been obtained for publication of this figure.

**Figure 6:**
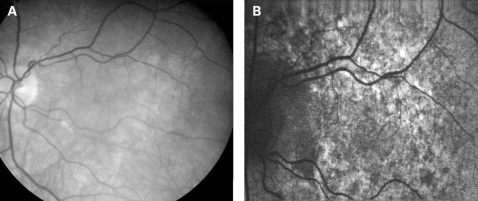
(A, B) Fundus photograph and AF image of patient 7. Note the diffuse abnormality of the macular AF demonstrated by speckled AF which is much greater than would be expected based on the fundus photograph. Patient consent has been obtained for publication of this figure.

The appearance on fundus autofluorescence (AF) imaging was of a diffuse macular abnormality. Decreased AF was present in areas of atrophy, and the pale deposits revealed increased AF. In the more common perifoveal atrophy phenotype, the retina surrounding the atrophic areas demonstrated speckled AF (figs 1, 3, 5). In the pattern dystrophy phenotype, there was a diffuse speckled appearance of the macular AF (figs 4 and 6). In neither case was the diffuse nature of the abnormality evident on biomicroscopy.

## DISCUSSION

**Figure 7:**
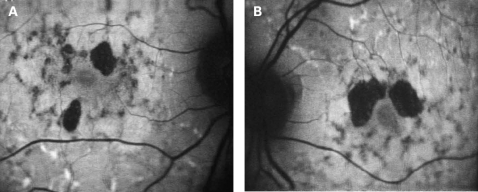
(A, B) AF images of Stargardt macular dystrophy. Note the areas of atrophy are within the fovea corresponding to decreased AF. There are surrounding areas of increased AF corresponding to the pale yellow flecks seen in STGD; between the flecks and atrophy, there is normal AF. Patient consent has been obtained for publication of this figure.

**Figure 8:**
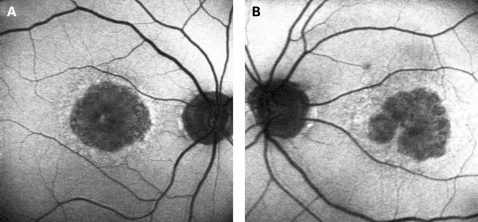
(A, B) AF images of geographic atrophy in age-related macular degeneration. The central areas of atrophy show decreased AF with a surrounding rim of increased AF. Patient consent has been obtained for publication of this figure.

The characteristics of AF associated with the A3243G mtDNA mutation are distinct and differ from the majority of other macular dystrophies. In Stargardt macular dystrophy (STGD), well-defined atrophy is associated with diminished AF and the flecks with increased AF. The intervening retina has homogeneous AF. Figure 7A,B shows AF imaging of a male with typical fundus findings of STGD. In pattern dystrophy, as with STGD, the areas of abnormal AF are limited to abnormal areas detectable by ophthalmoscopy. In geographic atrophy (GA) due to age-related macular degeneration (AMD), the atrophic area is associated with decreased AF and may have a rim of increased AF as described by Holz *et al* (fig 8).^20^ In each of these conditions, the AF abnormalities correlate with the funduscopic abnormalities, and there is no widespread speckled AF, as observed with the A3243G mtDNA mutation. In the latter, it is not only the area of clinically detectable disturbance that is abnormal; indeed the area of abnormal AF is significantly larger than would be expected from the funduscopic appearance.

**Figure 9:**
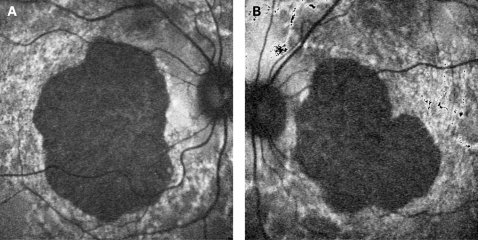
(A, B) AF images of a subject with the peripherin R172W mutation showing a diffuse speckled abnormality in macular autofluorescence, with a large well-demarcated central area of atrophy associated with reduced AF. Patient consent has been obtained for publication of this figure.

Perhaps the most difficult macular dystrophy to distinguish from that associated with the A3243G mtDNA mutation, by AF imaging alone, is the maculopathy caused by the dominant R172W peripherin mutation. The AF imaging in patients with the R172W mutation has been previously described and appears to depend on the stage of the disease.^21^ In the early symptomatic stages, these patients have a diffuse macular abnormality on AF consisting of speckled areas of increased and decreased AF within the macula simulating the pattern dystrophy-like phenotype of the A3243G mtDNA mutation. Later areas of atrophy develop within the areas of abnormal autofluorescence although not in the circular pattern seen in the patients with the mitochondrial dystrophy in this series. In addition, unlike A3243G maculopathy, the changes seen in R172W patients appear to be confined to the macular and peripapillary regions until very late in the disease when atrophic changes can extend beyond the arcades (fig 9).

The estimated prevalence of the A3243G mtDNA mutation resulting in MIDD in the diabetic population varies between 0.13 and 2.8%.^30^^–^38 In a recent multi-centre study, the systemic manifestations of MIDD in patients with diabetes were recorded in order to ascertain patients with diabetes who would benefit from screening for mitochondrial mutations.^29^ All patients included in this study had diabetes, 98% had bilateral neurosensory hearing loss, and 87% had macular pattern dystrophy. The percentage of patients with diabetes and deafness in this group was very high, as would be expected, given that patients were enrolled in the study based on these traits. In general, patients with the A3243G mtDNA mutation have a variety of disease manifestations including MIDD.^5^ ^6^ ^39^^–^41 Patients with macular dystrophy associated with the A3243G mutation diagnosed from an ophthalmology clinic may have more variable systemic manifestations than patients ascertained based on the presence of diabetes and deafness. In our small cohort of patients who presented with macular dystrophy, 83% (10/12) had symptomatic hearing loss, while only 33% (4/12) had diabetes. None of the patients in this series had diabetes without hearing loss. In addition, all patients in this series were diagnosed by the ophthalmologist as having a mitochondrial macular dystrophy, which was confirmed by testing for the A3243G mutation.

A potential weakness of this paper is the fact that only patients positive for the mitochondrial DNA mutation were included in the study. Although some patients were probably tested for the mutation and found to be negative, those patients were not included in this analysis. Therefore, we do not know the number of patients seen during the study period with a similar clinical phenotype who were negative for the A3243G mutation. Additionally, we assume the AF pattern we describe would be the same in all patients with a macular dystrophy secondary to the A3243G mutation. But because all of our patients were seen in an ophthalmology clinic, it might be interesting to perform a similar study on a population of diabetics known to have the A3243G mitochondrial DNA mutation to ascertain any differences in AF patterns in these patients.

The actual incidence of macular dystrophy associated with the A3243G mutation is probably not known, as most studies, including ours, have some selection bias based on the clinical setting from which the patients are ascertained. But certainly the incidence of macular dystrophy is probably much less common than the overall incidence of the A3243G mutation especially given the wide variety of clinical manifestations associated with this mutation.

Of interest for future study may be genotyping patients known to have the A3243G point mutation with and without evidence of macular dystrophy to determine if there is an increased incidence of genes known to increase AMD risk, such as complement factor H polymorphisms, in those patients with macular dystrophy compared with those without.

We conclude that the macular dystrophy associated with the A3243G mtDNA mutation has a recognizable phenotype by fundus autofluorescence in most cases and should be considered in the differential diagnosis, even in the absence of a personal or family history of diabetes and hearing loss.

## References

[1] van den Ouweland JM Lemkes HH Ruitenbeek W Mutation in mitochondrial tRNA^Leu(UUR)^ gene in a large pedigree with maternally transmitted type II diabetes mellitus and deafness. Nature Genet 1992;1:368–71 1284550 10.1038/ng0892-368

[2] Reardon W Ross RJM Sweeney MG Diabetes mellitus associated with a pathogenic point mutation in mitochondrial DNA. Lancet 1992;340:1376–9 1360090 10.1016/0140-6736(92)92560-3

[3] Kobayashi Y Momoi MY Tominaga K A point mutation in the mitochondrial tRNA(Leu)(UUR0 gene in MELAS (mitochondrial myopathy, encephalopathy, lactic acidosis and stroke-like episodes). Biochem Biophys Res Commun 1990;173:816–22 2268345 10.1016/s0006-291x(05)80860-5

[4] Goto Y Nonaka I Horai S A mutation in the tRNA^Leu(UUR)^ gene associated with the MELAS subgroup of mitochondrial encephalomyopathies. Nature 1990;348:651–3 2102678 10.1038/348651a0

[5] Manouvrier S Rötig A Hannebique G Point mutation of the mitochondrial tRNA^Leu^ gene (A 3243 G) in maternally inherited hypertrophic cardiomyopathy, diabetes mellitus, renal failure, and sensorineural deafness. J Med Genet 1985;32:654–6 7473662 10.1136/jmg.32.8.654 PMC1051645

[6] Chinnery PF Howell N Andrews RM Clinical mitochondrial genetics. J Med Genet 1999;36:425–36 10874629 PMC1734386

[7] Chinnery PF Howell N Lightowlers RN Molecular pathology of MELAS and MERRF The relationship between mutation load and clinical phenotypes. Brain 1997;120:1713–21 9365365 10.1093/brain/120.10.1713

[8] Hammans SR Sweeney MG Hanna MG The mitochondrial DNA transfer RNALeu(UUR) A→G(3243) mutation. A clinical and genetic study. Brain 1995;118:721–34 7600089 10.1093/brain/118.3.721

[9] Chinnery PF Howell N Andrews RM Mitochondrial DNA analysis: polymorphisms and pathogenicity. J Med Genet 1999;36:505–10 10424809 PMC1734403

[10] Mullie MA Harding AE Petty RKH The retinal manifestations of mitochondrial myopathy. Arch Ophthalmol 1985;103:1825–30 4074172 10.1001/archopht.1985.01050120059020

[11] Massin P Guillausseau P Bialettes B Macular pattern dystrophy associated with a mutation of mitochondrial DNA. Am J Ophthalmol 1995;120:247–8 7639309 10.1016/s0002-9394(14)72615-7

[12] Massin P Virally-Monod M Bialettes B Prevalence of macular pattern dystrophy in maternally inherited diabetes and deafness. Ophthalmology 1999;106:1821–7 10485557 10.1016/s0161-6420(99)90356-1

[13] Isashiki Y Nakagawa M Ohba N Acta Ophthalmol Scand 1998;76:6–13 9541428 10.1034/j.1600-0420.1998.760103.x

[14] Harrison TJ Boles RG Johnson DR Macular pattern retinal dystrophy, adult-onset diabetes, and deafness: A family study of A3243G mitochondrial heteroplasmy. Am J Ophthalmol 1997;124:217–21 9262546 10.1016/s0002-9394(14)70787-1

[15] Bonte CA Matthijs GL Cassiman JJ Macular pattern dystrophy in patients with deafness and diabetes. Retina 1997;17:216–21 9196933 10.1097/00006982-199705000-00008

[16] Andrews RM McNeela BJ Reading P Mitochondrial DNA disease masquerading as age-related macular degeneration. Eye 1999;13:595–6 10692944 10.1038/eye.1999.151

[17] Latkany P Ciulla TA Cucchillo P Mitochondrial maculopathy: geographic atrophy of the macula in the MELAS associated A to G 3243 mitochondrial DNA point mutation. Am J Ophthalmol 1999;128:112–14 10482110 10.1016/s0002-9394(99)00057-4

[18] Smith PR Bain SC Good PA Pigmentary retinal dystrophy and the syndrome of maternally inherited diabetes and deafness caused by the mitochondrial DNA 3243 tRNA^Leu^ A to G mutation. Ophthalmology 1999;106:1101–8 10366077 10.1016/S0161-6420(99)90244-0

[19] von Rückmann A Fitzke FW Bird AC In vivo fundus autofluorescence in macular dystrophies. Arch Ophthalmol 1997;115:609–15 9152128 10.1001/archopht.1997.01100150611006

[20] Holz FG Bellmann C Margaritidis M Patterns of increased in vivo fundus autofluorescence in the junctional zone of geographic atrophy of the retinal pigment epithelium associated with age-related macular degeneration. Graefes Arch Clin Exp Ophthalmol 1999;237:145–52 9987631 10.1007/s004170050209

[21] Downes SM Fitzke FW Holder GE Clinical features of codon 172 RDS macular dystrophy. Similar phenotype in 12 families. Arch Ophthalmol 1999;117:1373–83 10532447 10.1001/archopht.117.10.1373

[22] Downes SM Holder GE Fitzke FW Autosomal dominant cone and cone–rod dystrophy with mutations in the guanylate cyclase activator 1A gene-encoding guanylate cyclase activating protein-1. Arch Ophthalmol 2001;119:96–105 11146732

[23] Lois N Holder GE Bunce C Phenotypic subtypes of Stargardt macular dystrophy-fundus flavimaculatus. Arch Ophthalmol 2001;119:359–69 11231769 10.1001/archopht.119.3.359

[24] Bellmann C Jorzik J Spital G Symmetry of bilateral lesions in geographic atrophy in patients with age-related macular degeneration. Arch Ophthalmol 2002;120:579–84 12003606 10.1001/archopht.120.5.579

[25] Kurz-Levin MM Halfyard AS Bunce C Clinical variations in assessment of bull’s-eye maculopathy. Arch Ophthalmol 2002;120:567–75 12003605 10.1001/archopht.120.5.567

[26] Hammans SR Sweeney MG Brockington M Mitochondrial encephalopathies: molecular genetic diagnosis from blood samples. Lancet 1991;337:1311–13 1674297 10.1016/0140-6736(91)92981-7

[27] von Rückmann A Fitzke FW Bird AC Distribution of fundus autofluorescence with a scanning laser ophthalmoscope. Br J Ophthalmol 1995;79:407–12 7612549 10.1136/bjo.79.5.407 PMC505125

[28] Expert Committee on the Diagnosis and Classification of Diabetes Mellitus Report of the Expert Committee on the Diagnosis and Classification of Diabetes Mellitus. Diabetes Care 2003;26:5–20S 10.2337/diacare.26.2007.s5 12502614

[29] Guillausseau P Massin P Dubois-LaForgue D Maternally inherited diabetes and deafness: A multicenter Study. Ann Int Med 2001;134:721–8 11329229 10.7326/0003-4819-134-9_part_1-200105010-00008

[30] Vionnet N Passa P Froguel P Prevalence of mitochondrial gene mutations in families with diabetes mellitus. Lancet 1993;342:1429–30 7901716 10.1016/0140-6736(93)92792-r

[31] Newkirk JE Taylor RW Howell N Maternally inherited diabetes and deafness: prevalence in a hospital diabetic population. Diabetic Med 1997;14:457–60 9212310 10.1002/(SICI)1096-9136(199706)14:6<457::AID-DIA372>3.0.CO;2-W

[32] Kadowaki T Kadowaki H Mori Y A subtype of diabetes mellitus associated with a mutation of mitochondrial DNA. NEJM 1994;330:962–8 8121460 10.1056/NEJM199404073301403

[33] Kishimoto M Hashiramoto M Araki S Diabetes mellitus carrying a mutation in the mitochondrial tRNA^Leu(UUR)^ gene. Diabetologia 1995;38:193–200 7713314 10.1007/BF00400094

[34] Saker PJ Hattersley AT Barrow B UKPDS 21: Low prevalence of the mitochondrial transfer RNA gene (tRNA^Leu(UUR)^) mutation at position 3243bp in UK caucasian type 2 diabetic patients. Diabetic Med 1997;14:42–5 9017352 10.1002/(SICI)1096-9136(199701)14:1<42::AID-DIA295>3.0.CO;2-T

[35] t’Hart LM Lemkes HHPJ Heine RJ Prevalence of maternally inherited diabetes and deafness in diabetic populations in the Netherlands. Diabetologia 1994;37:1169–70 7867892 10.1007/BF00418385

[36] Holmes-Walker DJ Boyages SC Prevalence of maternally inherited diabetes and deafness in Australian diabetic subjects. Diabetologia 1999;42:1028–32 10491766 10.1007/s001250051264

[37] Otabe S Sakura H Shimokawa K The high prevalence of the diabetic patients with a mutation in the mitochondrial gene in Japan. J Clin Endocrinol Metab 1994;79:768–71 8077358 10.1210/jcem.79.3.8077358

[38] Lehto M Wipemo C Ivarsson S-A High frequency of mutation in MODY and mitochondrial genes in Scandinavian patients with familial early-onset diabetes. Diabetologia 1999;42:1131–7 10447526 10.1007/s001250051281

[39] Morgan-Hughes JA Sweeney MG Cooper JM Mitochondrial DNA (mtDNA) diseases: correlation of genotype to phenotype. Biochim Biophys Acta 1995;1271:135–40 7599199 10.1016/0925-4439(95)00020-5

[40] Deschauer M Wieser T Neudecker S Mitochondrial 3243 A G mutation (MELAS mutation) associated with painful muscle stiffness. Neuromusc Disord 1999;9:305–7 10407850 10.1016/s0960-8966(99)00019-x

[41] Chinnery PF Brown DT Archibald K Spinocerebellar ataxia and the A3243G and A8344G mtDNA mutations. J Med Genet 2002;39:e22 12011163 10.1136/jmg.39.5.e22 PMC1735128

